# Integrating Prime Editing and Cellular Reprogramming as Novel Strategies for Genetic Cardiac Disease Modeling and Treatment

**DOI:** 10.1007/s11886-024-02118-2

**Published:** 2024-09-11

**Authors:** Bing Yao, Zhiyong Lei, Manuel A. F. V. Gonçalves, Joost P. G. Sluijter

**Affiliations:** 1 Experimental Cardiology Laboratory, Department of Cardiology, Division of Heart and Lungs, University Medical Center Utrecht, Utrecht, The Netherlands; 2 Regenerative Medicine Center Utrecht, Circulatory Health Research Center, University Medical Center Utrecht, University Utrecht, Utrecht, The Netherlands; 3 CDL Research, University Medical Center Utrecht, Utrecht, The Netherlands; 4 Department of Cell and Chemical Biology, Leiden University Medical Centre, Leiden, The Netherlands

**Keywords:** Genetic cardiac diseases, CRISPR-based gene editing, Prime editing, Cellular reprogramming, Gene therapy

## Abstract

**Purpose of review:**

This review aims to evaluate the potential of CRISPR-based gene editing tools, particularly prime editors (PE), in treating genetic cardiac diseases. It seeks to answer how these tools can overcome current therapeutic limitations and explore the synergy between PE and induced pluripotent stem cell-derived cardiomyocytes (iPSC-CMs) for personalized medicine.

**Recent findings:**

Recent advancements in CRISPR technology, including CRISPR-Cas9, base editors, and PE, have demonstrated precise genome correction capabilities. Notably, PE has shown exceptional precision in correcting genetic mutations. Combining PE with iPSC-CMs has emerged as a robust platform for disease modeling and developing innovative treatments for genetic cardiac diseases.

**Summary:**

The review finds that PE, when combined with iPSC-CMs, holds significant promise for treating genetic cardiac diseases by addressing their root causes. This approach could revolutionize personalized medicine, offering more effective and precise treatments. Future research should focus on refining these technologies and their clinical applications.

## Introduction

In 2019, cardiovascular diseases (CVDs), including those caused by genetic and non-genetic factors, accounted for 17.9 million deaths worldwide. Genetic cardiac diseases, characterized by their familial inheritance patterns, lead to critical health issues, including heart failure, fatal arrhythmias, and sudden cardiac death [1, 2]. These diseases present significant health risks and impose broad socio-economic and psychological challenges, exacerbating stress, diminishing quality of life, and straining the financial resources of patients and their families. Current treatments primarily focus on symptom management, including both Implantable Cardioverter Defibrillator (ICD) and mechanical assistance devices [3], with heart transplantation being the only curative option in clinical practice. However, the limited availability of donor hearts makes waiting for a transplantation a significant challenge for patients. Given the complexity, difficulty in diagnosis, and limitations of current treatments for these genetic cardiac diseases, there is a demand for the development of precise and innovative therapeutic approaches that can fundamentally address the underlying causes of these disorders.

Clustered Regularly Interspaced Short Palindromic Repeats (CRISPR)-based gene editing represent a revolutionary set of methods that allow for targeted DNA manipulation of cells, plants, and animals, whose genetic change range includes insertions and deletions of varying sizes as well as specific base-pair substitutions [4, 5]. In the past decade, CRISPR-based gene editing tools, including different variant technologies like engineered CRISPR-Cas9 nucleases, base editors (e.g., cytidine and adenine base editors), and prime editors (PE), have been applied in cells, plants, and animals, offering as a result potentially effective treatment options for genetic cardiac diseases [6]. PE stands out for its precision and versatility, providing the potential for correcting a wide range of genetic anomalies with minimal off-target effects [7].

Reprogramming somatic cells into induced pluripotent stem cells (iPSCs) and subsequently differentiating these reprogrammed cells into cardiomyocytes (iPSC-CMs) provides a robust “disease-in-a-dish” platform for modeling genetic cardiac diseases and exploring candidate therapeutic treatments such as those involving the screening and testing of small-molecule drugs or gene editing reagents [8, 9]. Combined with PE-mediated genome editing, this cellular reprogramming approach acquires enhanced capabilities for disease modeling as well as opportunities for addressing these genetic cardiac diseases. This review summarizes recent studies on CRISPR-based gene editing tools for directly modeling genetic cardiac diseases and explores their integration with reprogramming technologies. We propose that combining PE with iPSC-CM technologies offers novel perspectives for both the modeling and potentially treatment of genetic cardiac diseases.

## CRISPR-Based Gene Editing Tools

### CRISPR-Cas9

Engineered CRISPR-associated protein 9 (Cas9) nucleases form a powerful set of genome editing tools that originated from a natural bacterial defense mechanism against phage infections [10]. These CRISPR-Cas9 nucleases consist of a single guide RNA (gRNA)-Cas9 complex. The gRNA-Cas9 complex first recognizes short DNA tracts named protospacer adjacent motifs (PAM) via the PAM-interacting domain of the Cas9 protein. In the case of the prototypic Cas9 nuclease from *Streptococcus pyogenes*, the PAM reads as NGG where N stand for any nucleotide. Next, the gRNA, formed by linking an invariant trans-activating RNA to a sequence-tailored CRISPR RNA, anneals with specific DNA sequences of typically 20 base pairs (protospacer) complementary to its 5’ end (spacer). Complementarity between RNA spacer and DNA protospacer sequences triggers conformational changes of the two Cas9 nuclease domains (i.e., HNH and RuvC) that ultimately lead to catalytic activation and double-stranded DNA break (DSB) formation 3-bp away from the PAM. Once the DSB is induced, cellular DNA repair mechanisms are activated with, among these, non-homologous end joining (NHEJ) [11], homology-directed repair (HDR) [12], and microhomology-mediated end joining (MMEJ) [13–16], are being actively exploited for genome editing purposes, e.g., for generating gene knockouts or gene knock-ins. The latter procedures require delivering into target cells DSB-repairing exogenous donor DNA templates encoding the edit(s) of interest.

### Base Editing

Beyond genome editing based on engineered CRISPR-Cas9 nucleases, base editing is a technology that permits introducing single base-pair substitutions within a gRNA-defined target site without inducing DSBs and independent of donor DNA usage. Typically, base editor proteins consist of a catalytically disabled version of a Cas9 enzyme that trigger single-stranded DNA breaks, or nicks, rather than intrinsically mutagenic DSBs [17–19]. Fusing these Cas9 nickases to effector moieties in the form of cytidine deaminases and adenosine deaminases, yields cytidine base editors (CBEs) and adenine base editors (ABEs), respectively [20]. Specifically, after gRNA-BE complex target site engagement, DNA denaturation and single-stranded DNA formation, facilitates local deamination by the effector domains. In the case of CBEs, cytosine (C) is deaminated into uracil (U) and via subsequent DNA repair or replication a target C·G base pair is converted into a T·A base pair. ABEs in turn deaminate adenine (A) into inosine (I) with the resulting base pair intermediates being subsequently converted via DNA repair or replication into a G·C base pair. Various CBE and ABE systems have been developed, each of which offering different editing windows and efficiencies [17, 18, 21–23].

### Prime Editing

PE is an advanced genome editing technology introduced at the end of 2019 [24] that offers increased precision and flexibility over that achieved with BEs and, importantly, with fewer undesired on-target and off-target effects when compared to those resulting from programmable nucleases, RNA-guided or otherwise. It is described as a “search-and-replace” method for precise DNA manipulations, mediating insertions, deletions, and all 12 base-pair conversions requiring in the process neither donor DNA templates nor DSBs [24].

#### The Development of Prime Editing

PE1 is the original prime editing system, consisting of a prime editor protein and a prime editing gRNA (pegRNA), which besides the spacer and scaffold sequences of regular gRNAs it has (i) a primer binding site (PBS) that hybridizes with the DNA flap generated after target DNA nicking, (ii) the edit of interest, and (iii) a reverse transcriptase template (RTT). This prime editor is a fusion between the *Streptococcus pyogenes* Cas9 H840A nickase and the wild-type reverse transcriptase (RT) from the Moloney murine leukaemia virus (MMLV). An optimized PE2 variant was generated by substituting the wild-type for an engineered MMLV RT variant whose five mutations improve thermostability, RNA–DNA template affinity and DNA synthesis processivity [24]. Similar to the gRNA-Cas9 complex, the pegRNA-prime editor complex binds first to the PAM associated with the intended genomic target site and, after spacer-protospacer hybridization, nicking of the PAM-containing DNA strand by the Cas9.H840A moiety yields a 3’-ended flap that anneals with the PBS of the pegRNA. Subsequently, the annealed product creates a primer for RT-mediated DNA synthesis over the RTT sequence and encoded edit of interest, resulting in a 3' DNA flap that anneals to the complementary genomic DNA. Finally, through endogenous DNA repair mechanisms, the edit of interest is permanently installed at the genomic target site completing the prime editing process [24]. With the further demand for efficient editing, various PE systems have been developed in recent years as shown in Table 1 [25].

**Table 1:**
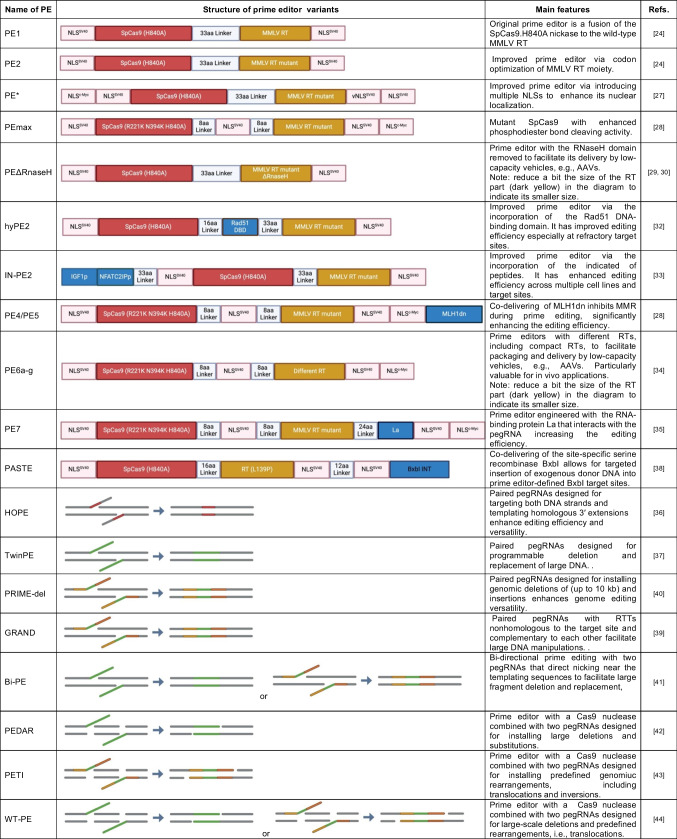
Overview of representative prime editing systems and their main attributes

Pink, NLS (Nuclear Localization Signal). Red, SpCas9 (Streptococcus pyogenes Cas9) and its variants. Light Blue, amino acid (aa) linkers. Brown, MMLV RT (Moloney murine leukemia virus reverse transcriptase) and its variants. Blue, other extra components, Rad51 DBD (DNA Binding Domain), IGF1p (Insulin-like Growth Factor 1 peptide), NFATC2IPp (Nuclear Factor of Activated T-Cells 2 Interacting Protein peptide), MLH1dn (dominant-negative variant of the mismatch repair protein MLH1) , La (La RNA-binding protein), BxbI  (BxbI serine site-specific recombinase)

Red segments: homologous sequences. Green segments: heterologous sequences. Orange and yellow segments: homologous sequences with different sequences

(Adapted with permission of Nature Research, from Chen PJ, Liu DR [25]; permission conveyed through Copyright Clearance Center, Inc.)

Beyond the aforementioned PE2 construct bearing an optimized MMLV RT region for improving the efficiency of reverse transcription [24], further and fast developments are yielding novel PE variants that include PE*, PEmax, and PEΔRnaseH. As previously shown for *S. pyogenes* Cas9 nucleases [26], prime editing can profit from the addition of nuclear localization signals (NLSs). For instance, PE* has enhanced nuclear localization, hence performance, owing to the addition of two extra NLSs to the both termini of PE2 [27]. PEmax has in turn a codon-optimized RT sequence and two additional mutations in its Cas9.H840A moiety for increased nicking activity [28]. Finally, via the removal of the prime editing-dispensable RNaseH domain, PEΔRNaseH displays a reduced size while maintaining editing efficiency [29, 30], which permits its delivery via carriers with limited cargo capacity, e.g., commonly used adeno-associated viral vectors [31].

In addition to optimizing the construction of PE proteins, adding extra components can further enhance their capabilities. For instance, HyPE2 incorporates the Rad51 DNA-binding domain that is hypothesized to promote DNA/RNA hybrid formation by binding to ssDNA and RNA and, in doing so, enhancing reverse transcription during prime editing. The HyPE2 construct improves PE efficiency by a median of 1.5-fold across various genomic sites and is particularly effective in genomic loci where PE2 demonstrates lower than 1% editing efficiency, achieving significant improvements at up to 34% of target sequences [32].

IN-PE2 enhances prime editing by including dual peptides, NFATC2IP and IGF1, thereby increasing prime editing outcomes across various cell lines and target sites. Velimirovic and co-researchers constructed two constructs, IN-GFP-PE2 and CTRL-GFP-PE2, and found that mESCs possess 1.58 fold higher amounts of IN-GFP-PE2 than of CTRL-GFP-PE2 with degradation occurring at a similar rate. These observations suggested that the two additional peptides increase either transcription or translation of the PE2 enzyme, offering an explanation for the increased activity of IN-PE2 [33]. The PE modality dubbed PE3 builds upon the PE2 system via the addition of a gRNA to direct the induction of another nick on the non-edited strand. This secondary gRNA-directed nick locates at an offset position from the primary nick directed by the pegRNA, enhancing in the process of editing efficiency by promoting the newly edited strand to serve as a template for DNA repair. PE3 and PE3b differ on the location of the additional secondary nick. In particular, in the latter approach, secondary nicking can only take place after edit incorporation as to minizine DSB formation via concomitant nicking of top and bottom DNA strands. Indeed, the PE3 modality can significantly enhance editing efficiency but, typically, it increases the rate of indels due to non-homologous end joining repair of DSBs created by concomitant nicking of top and bottom DNA strands, researchers thus need to consider the balance between editing efficiency and side-effects when selecting specific PE modalities [24]. The PE4 and PE5 systems build on PE2 and PE3 components, respectively, by incorporating a mismatch repair (MMR)-inhibiting protein consisting of a dominant-negative form of the human MLH1 protein (MLH1dn). By temporarily suppressing the cellular MMR pathway, MLH1dn enhances editing efficiency as this pathway tends to resolve mismatches in heteroduplex prime-editing intermediates consisting of edited and unedited strands [28].

Through Phage-Assisted Continuous Evolution (PACE) and protein engineering, smaller prime editor variants (516–810 bp coding sequences) were obtained, capable of yielding an editing efficiency improvement of up to 22-fold [34].The PE6 series employs PACE to significantly enhance the compactness of prime editing compared to PEmax and PEΔRnaseH.The PE6a-g variants have improved delivery vehicle compatibilities and editing efficacy in vivo, with one variant achieving a 24-fold improvement in loxP insertion efficiency in the murine brain cortex [34]. The PE7 system incorporates the RNA-binding protein La to enhance the interaction with pegRNAs, improving overall editing outcomes [35]. These diverse prime editors provide various options for achieving heightened genome editing efficiencies in different experimental contexts.

The development of paired or dual prime-editing systems, involving the use of two pegRNAs, represents a pivotal set of technologies offering precise and more versatile prime editing options. Indeed, these systems leverage the strengths of prime editing by incorporating dual pegRNAs that, by working in concert, expand the range of feasible genetic modifications from single base-pair substitutions and small insertions and deletions to larger-scale chromosomal edits. For instance, the Homologous 3′ Extensions Mediated Prime Editor (HOPE) uses paired pegRNAs encoding the same edits on both reverse transcribed DNA strands achieving efficient editing and improved product purity over that obtained with the PE3 system [36]. TwinPE employs two pegRNAs to template the synthesis of complementary DNA flaps on opposing strands of genomic DNA, enabling the programmable replacement or excision of DNA sequences at endogenous sites without double-strand breaks. TwinPE can also be combined with site-specific serine recombinases for targeted integration of large donor DNA into recombinase recognition sequences programmed by dual pegRNAs and, thereby, expand the range of precision exogenous gene insertion strategies. For instance, the combination of TwinPE and BxbI, a serine recombinase, successfully inserted a 5.6 kb DNA sequence into three genomic loci, exhibiting an editing efficiency of 2.5–6.8% [37]. Similarly, by fusing Cas9, reverse transcriptases and large serine integrases, Programmable Addition via Site-specific Targeting Elements (PASTE) achieves targeted gene insertions at efficiencies of ~ 4–5% for large cargos in primary human hepatocytes and T cells [38]. Moreover, GRAND, PRIME-Del, and Bi-PE employ a pair of pegRNAs with reverse transcription templates complementary to each other that are nonhomologous to the target DNA [39–41]. PRIME-Del allows for deletions of up to 10 kb with significantly higher precision and fewer unintended off-target effects than that resulting from using the CRISPR-Cas9 system [40]. PE-Cas9-based deletion and repair (PEDAR) uses dual pegRNAs and a regular Cas9 nuclease fused to a reverse transcriptase for creating large genomic deletions and for replacing DNA fragments (1-10 kb) with an intended exogenous sequence (up to 60 bp). PEDAR was used in a tyrosinemia I mouse model derived by replacing a 19-bp sequence with a ~ 1.3-kb neo-expression cassette at exon 5 of the* Fah* gene. In particular, Jiang and co-researchers designed two pegRNAs, aimed at deleting the large insertion and inserting the missing 19-bp* Fah *gene fragment. One week later, they detected a 0.76 ± 0.25% correction rate in PEDAR-treated mice, but no correction in Cas9-treated mice [42]. Additionally, equally building on fusion constructs between Cas9 nucleases and reverse transcriptases, prime editor nuclease-mediated translocation and inversion (PETI) and WT-PE were shown to be capable of generating large genomic deletions and defined chromosomal translocations with efficiencies comparable to that achieve with regular Cas9 nuclease [43, 44].

The continuous innovation in prime editing systems highlights the rapid advancements in the field of gene editing. Each system offers distinct advantages tailored to specific editing requirements, showcasing a remarkable diversity in established and potential applications that can in principle extend to therapeutic gene correction and multiplexing genetic engineering of multicellular organisms.

#### The Advantages of Prime Editing

PE represents a significant advancement in the genome editing field, offering distinct advantages over other CRISPR-based tools (see Table 2). Firstly, unlike CRISPR-Cas9-based gene editing, PE achieves DNA editing without creating DSBs, thereby minimizing the risk of undesired outcomes at on-target and off-target sites. Such outcomes include deletions, duplications and translocations at the DNA level and aneuploidy and chromothripsis at the cellular level [25]. Secondly, as it is more flexible, PE is applicable to a broader range of genetic targets and diseases, particularly those requiring multiple edits beyond simple base-pair substitutions [45]. The precision of PE is enhanced by its unique mechanism, which involves the innovative combination of RTT and PBS sequences in a single pegRNA that license specific hybridization steps between RNA and DNA templates. Prime editing offers unprecedented specificity owing to the required multitier complementarity between pegRNA sequences (i.e., spacer, PBS and RTT) and target DNA. These multiple hybridization requirements ensure high-fidelity base incorporation and accurate gene correction [24]. In summary, the versatility, precision, and reduced off-target effects offered by PE enhance the safety and efficacy prospects of these technologies for therapeutic applications.

**Table 2:**
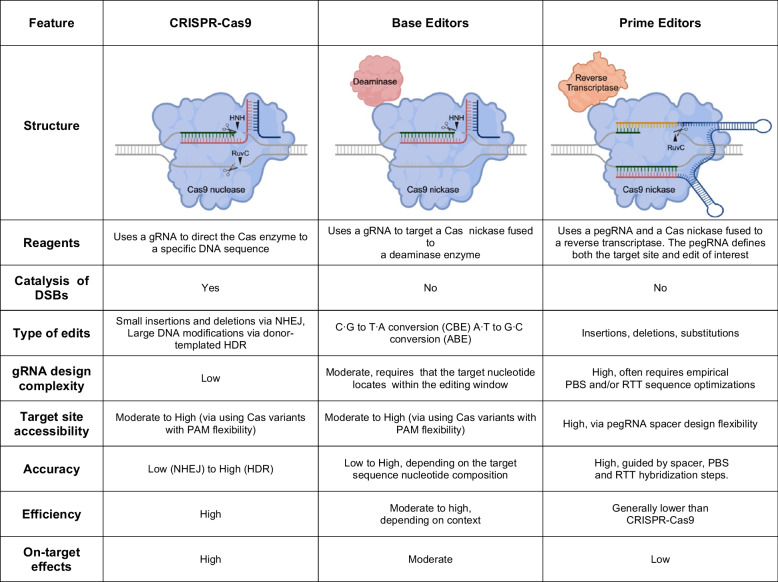
Comparison between CRISPR-Cas9, base editors, and prime editors

Green DNA represents the target sequences (protospacers) located next to the respective protospacer adjacent motif (PAM) sites (now indicated), red and blue sequences represent, respectively, the CRISPR RNA and trans-activating CRISPR RNA (normally fused, not shown here) of the gRNA with the portion hybridizing to the protospacer corresponding to the spacer sequence. In the case of prime editors, the gRNA is extended at the 3’ terminus with a prime binding site (PBS) and a reverse transcriptase template (RTT) encoding the edit of interest. This modified gRNA is named a prime editing gRNA (pegRNA)

The arrowheads indicated the RuvC and HNH  nuclease domains of Cas9. The scissors mark the positions of phosphodiester bond cleavage catalyzed by each of these nuclease domains

#### Current Applications of Prime Editing in CVD

PE has effectively corrected small insertions, deletions, and substitutions in various cell types. For correcting point mutation in the Duchenne muscular dystrophy (*DMD*) gene, PE efficiencies ranged from 21% to 38% in HEK293T cells and 22% in myoblasts [46–49]. Introduction of prime editing complexes via high-capacity adenovector particles can further enhance the performance of the editing process [50], including at defective *DMD* alleles in human myoblasts and iPSC-derived cardiomyocytes [51]. Additionally, a﻿ large-scale deletion in the *DMD* gene, spanning from exon 17 through 55, was successfully achieved using WT-PE [44]. 

Lipid nanoparticles (LNPs) delivering chemically-modified pegRNA and prime editor mRNA were used in HAP1 reporter cells to achieve an editing efficiency of 54% [52]. PE-mediated gene editing was successful in iPSC-CMs, achieving notable editing endpoints in cardiomyocytes. For instance, in *DMD* exon 51–deleted human iPSCs (ΔEx51 iPSCs), PE3-mediated modification of splice donor sites in the dystrophin gene was used to complete a -GT insertion [53]. This approach achieved gene editing efficiencies of up to 54%. After differentiation, the edited ΔEx51 iPSC-CMs were confirmed to have restored dystrophin protein expression when compared to control iPSC-CMs. In the case of the RBM20 R636S mutation, up to 40% correction was observed, releasing hypertrophic cardiomyopathy (HCM) symptoms [54].

PE has been applied in vivo through viral vector delivery methods. Adenoviral vectors (Advs) delivered PE3 components into a phenylketonuria mouse model yielding up to 11.1% editing efficiencies resulting in 2.0%-6.0% of the wild-type *Pah* enzyme activity in treated mice [55]. Moreover, Liu and colleagues developed a dual-AAV (adeno-associated viral vector) encoding a split-PE system that retains 75% editing activity of that achieved with the full-length PE construct [56]. These findings underscore the robust capabilities of prime editing for targeted and precise genetic modifications both in vitro and in vivo, opening up new possibilities for treating a wide range of genetic cardiac diseases.

## Genetic Cardiac Disease iPSC-CM Modeling by Reprogramming and Gene Editing

Some mutations of genetic cardiac diseases can lead to arrhythmias such as Long QT Syndrome (LQTS) and Short QT Syndrome (SQTS), as well as to structural abnormalities like HCM and dilated cardiomyopathy (DCM), arrhythmogenic cardiomyopathy (ACM). These conditions significantly increase the risk of heart failure and even sudden cardiac death. Developing models through reprogramming of human somatic cells into iPSCs and subsequently differentiate the resulting iPSCs into cardiomyocytes is a powerful approach for improving our understanding of the underlying genetic disease-causing mechanisms and, thereby, develop novel therapeutic approaches for these cardiac diseases [57–59]. By introducing a set of defined transcription factors (e.g., the initial “Yamanaka cocktail” Oct4, Sox2, Klf4, and c-Myc) into somatic cells, mainly skin fibroblasts or blood cells, triggers cellular reprogramming into iPSCs [60, 61]. These reprogrammed iPSCs possess pluripotency, enabling them to differentiate into any human cell type, including cardiomyocytes [62]. These iPSC-CM models not only aid in investigating cardiac diseases caused by specific genetic mutations, and provide for cellular substrates for drug screening and therapy development. They also overcome the technical and ethical limitations of embryonic stem cell (ESC) use in animal model research and therapeutic development [57, 63].

### Patient-derived iPSC-CM Models

Patient-derived iPSC-CMs, which carry the genetic information of the donor, exhibit cardiac disease phenotypes, effectively creating disease-in-a-dish systems that enable investigations into the causes of these diseases. For example, the main characteristic of LQTS is a prolonged QT interval on the electrocardiogram (ECG) [64, 65], that increases the risk of sudden cardiac death. Models of iPSC-CMs from patients with mutations in the *NAA10* gene have recreated the LQTS phenotype [66]. Electrophysiological studies have shown prolonged action potential duration (APD) and corrected field potential duration. Researchers have used ICaL blockers to correct the prolonged field potential duration in patient-derived iPSC-CMs, demonstrating their effectiveness as therapeutic models. Similarly, treating iPSC-CMs from patients carrying the *KCNQ1/TRPM4 *double mutations with verapamil and lidocaine significantly shortened the QT interval [67]. Conversely, SQTS is characterized by an abnormally short QT interval on the ECG, leading to fainting, palpitations, atrial and ventricular arrhythmias, and sudden cardiac death [68]. iPSC-CMs carrying the T618I mutation in *KCNH2* successfully mimic the clinical manifestations of SQTS, such as shortened action potential duration and abnormally short QT intervals [69].

For cardiac diseases caused by structural abnormalities, tractable patient-derived iPSC-CM models have been developed. HCM is one of the most common genetic cardiac diseases, characterized by abnormal thickening of the left ventricular wall and ventricular septum, leading to heart failure, arrhythmias, and a high risk of sudden death [70]. Patient-derived iPSC-CMs with the *MYL2*-R58Q mutation were 30% larger than control iPSC-CMs at day 60, exhibited disarray in myofibrils, and had a higher percentage of irregularly beating cells, thereby accurately representing the HCM phenotype with reduced calcium transients [71]. In 2022 and 2023, iPSC-CMs with other HCM mutations like *MYBPC3* R326Q [72], *TNNT2 *Δ160E [73], *JPH2* Thr161Lys [74], and *RAF1* [75] also exhibited abnormal calcium handling, leading to increased intracellular calcium concentrations. DCM is characterized by left ventricular or biventricular dilation and impaired systolic function, which occurs in the absence of external causes like hypertension, valvular, congenital, or ischemic heart disease [76, 77]. Studies on mutations *TNNT2*-R173W [78], and *TNNT2*-R92W [57] have shown that these disruptions interfere with the interactions within the troponin-tropomyosin complex and impair protein kinase A (PKA) binding to sarcomeric microdomains, thereby affecting calcium handling and contractility. Another iPSC-CMs model with mixed DCM/ACM phenotypes carries the *PLN* p.Arg14del mutation, which exhibit disrupted regulation of the sarcoplasmic/endoplasmic reticulum Ca^2^⁺-ATPase (SERCA2a), impairing calcium handling in cardiomyocytes [79]. Besides successfully replicating cardiac disease phenotypes for hypotheses-driven mechanistic studies, patient-derived iPSC-CM models are also facilitating unbiased high-throughput drug screens using large small-molecule libraries. However, there are some limitations with these models as the maturation status of the obtained iPSC-CMs can significantly hamper definitive conclusions as they normally display gene expression programs of fetal instead of bona fide adult or mature cardiomyocytes. In this regard, longer culture periods, mechanical and electrical stimulation, organoid assemblies, and the use of scaffolds, and exposure to defined small-molecule cocktails have, to different extents, shown to provide for further maturation of iPSC-CMs in vitro. Additionally, challenges include obtaining cells from patients with rare mutations or in determining how specific genetic abnormalities may lead to previously unidentified diseases.

### Gene Edited Healthy Donor-derived iPSC-CM Models

To better establish genotype-phenotype associations, a new approach involves applying gene editing technologies to edit healthy donor-derived iPSCs and, in doing so, create isogenic pairs of mutant and wild-type iPSC-CMs that share the same genetic background. Indeed, unlike patient-derived iPSC-CM models, gene editing allows for the installation of specific mutations within normal, healthy iPSCs. This approach enables the creation of models unrestricted by patient-specific disease conditions, facilitating the construction of genetic mutation-specific disease models, including their isogenic controls, and providing a new platform to study the role and effects of SNPs, structural variants and mutations in specific *loci*. Recent applications of CRISPR-Cas9 technologies have introduced mutations associated with the *CACNA1C *[80], *KCNH2 *[81] and *hERG* genes [82] into healthy donor-derived iPSC-CMs, inducing LQTS1 and LQTS2. These models successfully replicated the LQTS phenotypes, including action potential prolongation and early afterdepolarizations (EADs), without altering the overall genomic expression profile, thereby validating the effectiveness and precision of genome editing techniques. Moreover, CRISPR-Cas9 has been used to investigate specific mutations in DCM. One study focused on the *BAG3* R477H mutation, associated with DCM, and used CRISPR-Cas9 to introduce this mutation into iPSC-CMs derived from healthy donors [83]. This approach allowed researchers to examine the impact of the mutation on cellular structures and functions without the confounding effects of additional genetic variations that might be present in patient-derived cells. Similarly, a mutation *TnT*-R173W, known to destabilize interactions within the sarcomeric troponin-tropomyosin complex and affect heart contraction mechanics, was studied using CRISPR-Cas9 technology [78]. Gene edited iPSC-CMs derived from healthy donors displayed the same phenotype as that observed in patient-derived iPSC-CM models, offering flexible capabilities to directly study the impact of defined genetic alterations on cardiomyocyte function.

## Genetic Cardiac Disease iPSC-CM Treatment by Gene Editing

With the rapid advancement of gene editing tools, e.g., CRISPR-Cas9, CBE, ABE, and PE, it has become realistic to treat genetic cardiac diseases in vivo via direct correction or modification of endogenous genes. Indeed, these tools offer a potential therapeutic approach by precisely correcting or altering specific genetic mutations that, as a result, significantly improve cardiac function and prolong life. In patient-derived iPSC-CM models, ABEs have achieved gene correction rates of over 90% for mutations such as *RBM20* R634Q and *MYH7 *R403Q [84], indicating that gene editing is a very promising approach for treating genetic cardiac diseases [54]. The observed successes were extended beyond cellular studies to animal models. A dual-AAV delivered ABE system has demonstrated efficient editing of several mutations associated with HCM, including *Lmna *c.1621C > T [85], *Rbm20* R634Q [54], *Myh6 *c.1211C > T [86], *Myh6 *R403Q [87], and *MYH7 *c.1208G > A (R403Q) [84]. In mouse models, this type of genetic intervention not only achieved high editing efficiency but also resulted in extended lifespans and release of cardiac symptoms, hence effectively correcting simultaneously both the genotype and pathogenic phenotypes.

## The Integration of Prime Editing and Reprogramming for Genetic Cardiac Disease

**Fig. 1:**
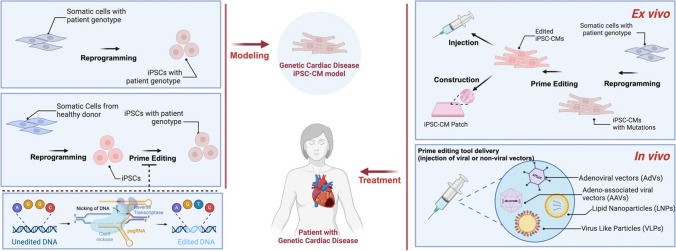
The integration of cellular reprogramming and prime editing in genetic cardiac disease modeling (**left**) and treatment (**right**)

The integration of PE and cellular reprogramming technologies represents a pivotal achievement in the field of genetic cardiac disease modeling and treatment. This innovative approach combines the high precision of PE in establishing targeted genetic alterations with the versatility of iPSC systems, creating as a consequence powerful platforms for mechanistic research and clinical treatment of these genetic cardiac diseases (see Fig. 1).

 Prime editing of healthy donor-derived iPSC-CMs offers a flexible disease modelling approach especially in view of the aforementioned challenges associated with the exclusive use of patient-derived iPSC-CMs. In particular, the incomplete maturation status of iPSC-CMs under most culture conditions, the rare nature of certain genetic diseases, and the difficulty in understanding the disease mechanisms in different genetic backgrounds. For treatment, both ex vivo and in vivo prime editing strategies are possible based on previous studies [84, 85, 87–90]. The former involves harvesting cells from patients, reprogramming them into iPSCs, and then correcting the mutations by prime editing before introducing corrected iPSC-CMs into the patient. This approach not only ensures that the modified cells are free from genetic defects but also reduces the risk of immunological rejection, offering a personalized therapeutic option. In vivo strategies explore the direct application of prime editing within the body of the patient. This approach employs advanced delivery systems to introduce genetic modifications directly in cells from the affected cardiac tissues. Together, these strategies enhance the appeal of using iPSC-CMs in conjunction with prime editing for flexible disease traits modeling and correction.

## Challenges of Prime editing in Genetic Cardiac Disease Treatment

Prime editing is a powerful gene editing technology, offering higher precision and fewer byproducts like undesired insertions or deletions. However, despite its potential, prime editing applications in genetic cardiac disease treatments remains restricted by several limitations, particularly in the aspects pertaining to the editing efficiency, off-target effects, off-target organ editing, and delivery efficacy.

 Previous studies have demonstrated notable editing efficiency of PE in neonatal mice [55], a recent study evaluating editing efficiencies across 54,836 pegRNAs showed an editing efficiency of around 20%, with efficiencies decreasing in primary cells [91]. Additionally, the editing efficiency of PE is influenced by both the target loci and the specific cell types. For instance, the efficiencies of PE-mediated installation of point mutations and small fragment insertions at the HEK3 locus have demonstrated in various cell lines, including HEK293FT cells, K526 cells, U2OS cells, and HeLa cells [24]. Although PE is more precise than CRISPR-Cas9 nucleases, off-target effects at the genome and transcriptome levels will have to be carefully assessed in each individual clinical settings [24]. As aforementioned, it is also crucial that the prime editor and pegRNA operate in an efficient and precise manner at the intended targeted organ. Current delivery systems often lack the precision required to limit action to the target organs, which can lead to uncontrollable effects in other organs. To address this issue, the development of delivery vectors prioritized for heart tissue and the use of heart-specific promoters are promising strategies [92–94]. The delivery efficiency of prime editing components into cells is another factor influencing editing efficiency. The components of PE are larger and more complex than those used in CRISPR-Cas9 and BEs [13, 17, 18, 24]. This complexity makes it difficult to package and deliver these components efficiently, particularly when using commonly used viral vectors, e.g., AAVs which have their packaging size limitations [29, 30, 56, 95]. Alternative viral vectors such as high-capacity adenovectors and baculoviral vectors possess the payload capacity for delivering all PE components in single particles in an efficient manner [50, 96, 97]. Moreover, non-viral delivery methods, such as LNPs and Virus-Like Particles (VLPs), are being explored but generally present lower delivery efficiencies [52, 98].

## Conclusions

The integration of iPSC-CM and PE technologies represents a potential approach for genetic cardiac diseases, offering dual advantages of personalized disease modeling and creating therapeutic treatments. In addition, these technologies provide a robust platform for displaying disease mechanisms and drug screening, reflective of patient-specific cardiac phenotypes. Indeed, prime editing enhances disease modeling and treatment by allowing precise genomic modifications without the off-target effects and limitations of CRISPR-Cas9 and BEs.

However, the integration of PE and iPSC-CM technologies is, clearly, not without limitations. Chiefly amongst these, the variability regarding the efficiency and specificity of somatic cell reprogramming and subsequent iPSC differentiation into mature cardiomyocytes potentially affects the consistency and reproducibility of disease models. Moreover, the efficiency and specificity of prime editing can equally greatly vary depending on the target loci and cell types of interest. Importantly, these limitations are being addressed through the development and implementation of novel iPSC differentiation protocols [99], and PE delivery systems that, in in vivo contexts, should ideally display target-organ specificity.

## Data Availability

No datasets were generated or analysed during the current study.
